# Identification and validation of lncRNAs involved in m6A regulation for patients with ovarian cancer

**DOI:** 10.1186/s12935-021-02076-7

**Published:** 2021-07-08

**Authors:** Jianfeng Zheng, Jialu Guo, Benben Cao, Ying Zhou, Jinyi Tong

**Affiliations:** 1 Department of Obstetrics and Gynecology, Affiliated Hangzhou Hospital, Nanjing Medical University, Hangzhou, 310008 Zhejiang Province China; 2 Department of Obstetrics and Gynecology, Hangzhou Women’s Hospital, Hangzhou, 310008 Zhejiang Province China; 3 Department of Clinical Pharmacology, Key Laboratory of Clinical Cancer Pharmacology and Toxicology Research of Zhejiang Province, Affiliated Hangzhou First People’s Hospital, Zhejiang University School of Medicine, Hangzhou, 310008 Zhejiang Province China; 4 Department of Fourth Clinical Medical College, Zhejiang Chinese Medical University, Hangzhou, 310006 Zhejiang Province China

**Keywords:** Ovarian cancer, N6-methyladenosine modification, lncRNA, Risk score model, Cell function assays

## Abstract

**Background:**

Both N6-methyladenosine (m6A) modification and lncRNAs play an important role in the carcinogenesis and cancer inhibition of ovarian cancer (OC). However, lncRNAs involved in m6A regulation (LI-m6As) have never been reported in OC. Herein, we aimed to identify and validate a signature based on LI-m6A for OC.

**Methods:**

RNA sequencing profiles with corresponding clinical information associated with OC and 23 m6A regulators were extracted from TCGA. The Pearson correlation coefficient (PCC) between lncRNAs and 23 m6A regulators (|PCC|> 0.4 and p < 0.01) was calculated to identify LI-m6As. The LI-m6As with significant prognostic value were screened based on univariate Cox regression analysis to construct a risk model by LASSO Cox regression. Gene Set Enrichment Analysis (GSEA) was implemented to survey the biological functions of the risk groups. Several clinicopathological characteristics were utilized to evaluate their ability to predict prognosis, and a nomogram was constructed to evaluate the accuracy of survival prediction. Besides, immune microenvironment, checkpoint, and drug sensitivity in the two risk groups were compared using comprehensive algorithms. Finally, real-time qPCR analysis and cell counting kit-8 assays were performed on an alternative lncRNA, CACNA1G-AS1.

**Results:**

The training cohort involving 258 OC patients and the validation cohort involving 111 OC patients were downloaded from TCGA. According to the PCC between the m6A regulators and lncRNAs, 129 LI-m6As were obtained to perform univariate Cox regression analysis and then 10 significant prognostic LI-m6As were identified. A prognostic signature containing four LI-m6As (AC010894.3, ACAP2-IT1, CACNA1G-AS1, and UBA6-AS1) was constructed according to the LASSO Cox regression analysis of the 10 LI-m6As. The prognostic signature was validated to show completely opposite prognostic value in the two risk groups and adverse overall survival (OS) in several clinicopathological characteristics. GSEA indicated that differentially expressed genes in disparate risk groups were enriched in several tumor-related pathways. At the same time, we found significant differences in some immune cells and chemotherapeutic agents between the two groups. An alternative lncRNA, CACNA1G-AS1, was proven to be upregulated in 30 OC specimens and 3 OC cell lines relative to control. Furthermore, knockdown of CACNA1G‐AS1 was proven to restrain the multiplication capacity of OC cells.

**Conclusions:**

Based on the four LI-m6As (AC010894.3, ACAP2-IT1, CACNA1G-AS1, and UBA6-AS1), the risk model we identified can independently predict the OS and therapeutic value of OC. CACNA1G‐AS1 was preliminarily proved to be a malignant lncRNA.

**Supplementary Information:**

The online version contains supplementary material available at 10.1186/s12935-021-02076-7.

## Background

Ovarian cancer (OC) is the main cause of gynecological cancer-related death worldwide [1]. Because of the lack of clinical manifestations in the early stage, 70% of patients are diagnosed in the middle or late stages [1]. It is also difficult to perform radical surgery, leading to a high OC mortality rate of OC [1]. OC is characterized by obvious tissue heterogeneity with genomic characteristics, which shows that it easily develops resistance to chemotherapy and leads to a high tumor recurrence rate [1]. Therefore, improving the prognosis of OC patients by exploring novel diagnoses and therapies is an urgent problem to address.

Studies have shown that genome-level epigenetic modifications, such as DNA methylation, histone modification and RNA editing, are crucial to tumorigenesis. N6-methyladenosine (m6A) modification was first detected in the poly (A) RNA component in 1974 and is considered to be the most abundant posttranscriptional modification of mRNA [2]. m6A modification is mainly associated with three types of proteases that serve as regulators, namely, methyltransferases (writers), demethylases (erasers), and signal transducers (readers) [2]. Numerous studies have shown that different levels of m6A regulators are associated with the self-renewal of tumor stem cells, the proliferation of cancer cells, and the sensitivity to chemotherapy [2]. A previous study showed that high-frequency genetic alterations of m6A RNA methylation regulators are crucial for the progression [3] and that m6A modification contributes to PARPi resistance in OC [4]. In OC, YTHDF1 (reader) facilitates the expression of EIF3C [5] and the stem cell-like phenotype of cisplatin resistance [6] in a m6A-dependent manner, thereby strengthening tumorigenesis and metastasis. ALKBH5 (eraser) [7] and YTHDF2 (reader) [8, 9] were shown to regulate the carcinogenesis of OC by modulating m6A levels. IGF2BP1 (reader) was demonstrated to augment the translation of serum response factor through m6A modification [10]. METTL3 (writer) can regulate m6A methylation and thus regulate the malignancy of OC [11–14]. It has been proven that FTO (eraser) regulates PDE4B and PDE1C by m6A and thus plays a tumor suppressor function in OC [15]. Hence, OC is to a large extent mediated by m6A modification; nevertheless, studies reporting the mechanism of m6A modification and its role in OC pathology remain unclear, which motivates us to explore m6A modification for the pathogenesis and therapeutic direction of OC.

lncRNAs are more than 200 nucleotides in length, do not have a protein-coding function and participate in carcinogenesis or cancer inhibition at both the transcriptional and posttranscriptional level in various cancers, including OC [16]. Recent reports have indicated that m6A is involved in the regulation of the physiological functions of known lncRNAs. M6A can regulate the structure for lncRNA via binding sites for m6A readers, which might allow specific RNA-binding proteins access to m6A residues [17, 18]. For instance, m6A acts as a structural switch of lncRNA MALAT1 by modulating its structure, which is associated with cancer malignancy [17, 19, 20]. In addition, METTL16 (writer) was confirmed as a triple-stranded RNA-binding protein of lncRNA MALAT1 [20, 21]. M6A can also affect the function of lncRNAs through the ceRNA network. For example, lncRNA FAM225A stabilized by m6A was identified to serve as a sponge for miR-590-3p and miR-1275 in nasopharyngeal carcinoma [22]. Similarly, LINC00958 was stabilized by METTL3 (writer), and upregulated LINC00958 was involved in the malignancy of hepatocellular carcinoma progression by sponging miR-3619-5p [23]. Hence, lncRNAs can be modified by m6A and m6A can regulate their function. It is very likely that lncRNAs involved in m6A regulation (LI-m6As) will provide new ideas in the search for cancer therapeutic targets. Nonetheless, research on LI-m6As in OC remains lacking.

In our study, we identified LI-m6As based on the Pearson correlation coefficient (PCC) between lncRNAs and 23 m6A regulators (|PCC|> 0.4), and we found ten LI-m6As with significant prognostic value from the TCGA dataset. An LI-m6A prognostic signature was developed based on four LI-m6As (ACAP2-IT1, CACNA1G-AS1, AC010894.3, and UBA6-AS1) which demonstrated an ability to stratify OC patients into low-risk and high-risk groups with adverse OS and it was verified both in the training cohort and validation cohort. We further conducted comprehensive analyses of this risk model. An alternative lncRNA, CACNA1G‐AS1, was preliminarily proven to be an oncogene of OC in vitro.

## Methods

### Datasets and preprocessing

The RNA sequencing profiles with clinical information were downloaded from TCGA (https://toil.xenahubs.net). The total OC samples were randomly divided into the training or validation cohort at a ratio of 3:7. Twenty-three m6A regulators were extracted from the TCGA database based on previous studies [24]. The lncRNAs were identified based on their annotation information in Genome Reference Consortium Human Build 38 (GRCh38) of the GENCODE database (https://www.gencodegenes.org/) [25]. The Pearson correlation coefficient (PCC) is important for exploring the relationships between variables. Therefore, PCC between lncRNAs and 23 m6A regulators (|PCC|> 0.4 and p < 0.01) was calculated to identify lncRNAs involved in m6A regulation (LI-m6As) [26].

### Bioinformatic analysis

The potential cis interaction between LI-m6As and mRNAs transcribed at the same chromosome within 200 kb was searched from UCSC dataset [27]. Networks based on overlapped TFs was further constructed to detect the trans regulating function of LI-m6As and m6A genes [28]. The miRNAs targeted by the corresponding LI-m6As were speculated by starBase and NPInter [29]. The target mRNAs by the corresponding miRNAs were speculated using miRTarBase and NPInter [29]. Subsequently, the ceRNA network based on the same miRNAs was constructed [29].

Univariate Cox regression analysis was performed on the candidate LI-m6As based on the survival package of R (http://bioconductor.org/packages/survivalr/) to filtrate LI-m6As with significant prognostic value (P < 0.05). Then, the LASSO Cox regression was performed by employing the glmnet R package to construct a risk model. The degree of Lasso regression complexity was controlled by the appropriate parameter λ, and λ was selected to build the model for accuracy in our study [30]. Ultimately, a prognostic signature was developed based on the following formula:

$$Risk\,score = \sum\limits_{i = 1}^n {\left( {Coe{f_i} \times Gen{e_i}} \right)}$$

In the formula, Coef_i_ represents the coefficients, and Gene_i_ is the FPKM value of each LI-m6A. OC patients in our study were divided into low-risk or high-risk groups according to the median of risk scores (RSs). Kaplan–Meier (K-M) survival analysis was performed by the log-rank test to draw a survival curve.

Several clinicopathological characteristics were utilized to evaluate their ability to predict prognosis. A nomogram based on multivariate Cox regression was constructed for visualization of the clinicopathology and RSs, which verified their accuracy by calibration plots [31].

For the biological functions of the risk groups, differentially expressed genes (DEGs) between the two risk groups were filtered on the basis of the limma R package to perform gene ontology (GO) function and KEGG pathway enrichment analysis using clusterProfiler R package [32]. Gene set enrichment analysis (GSEA) was implemented to survey the biological function of the risk groups using GSEA (version 4.0.3) software. The protein–protein interaction (PPI) network for identifying key modules was established using the MCODE plug-in of Cytoscape.

Moreover, we estimated the differences in the immune microenvironment and immune checkpoint genes between the risk groups by employing five algorithms (Estimate, ssGSEA, Cibersort, MCPcounter and xCell) [33, 34]. Using Tumor Immune Dysfunction and Exclusion (TIDE) dataset, the response to immune checkpoint blockade was also predicted [35] and the TIDE scores were compared between risk groups. The chemotherapy drugs were extracted from Genomics of Drug Sensitivity in Cancer (GDSC) database [36] and the half maximal inhibitory concentration (IC50) was computed by pRRophetic R package [37].

### In vitro assays

The specimens were collected from OC patients who had received no chemotherapy or radiotherapy prior to surgery after approval from the Ethics Committee of Hangzhou First People's Hospital. The OC tissues following resection were confirmed by at least two experienced pathologists based on the FIGO staging system.

The cell lines SKOV-3, HO-8910, A2780, and IOSE-80 were purchased from iCell Bioscience Inc (Shanghai, China) and incubated in an incubator with 5% CO_2_ at 37 °C. The composition of the media was 89% RPMI 1640 media with 10% fetal bovine serum and 1% penicillin–streptomycin. The siRNAs used for transfection were established by GenePharma (Shanghai, China), and OC cells were transfected with siRNAs using jetPRIME® transfection reagent (Polyplus Transfection, China). The sequences of the siRNAs we used are shown in Table 1.

**Table 1:** siRNA and primer sequences

siRNA	Sense (5'-3')	Antisense (5'-3')
siRNA-1	GCAGACAAAUGGACAACAUTT	AUGUUGUCCAUUUGUCUGCTT
siRNA-2	GCCUUCGCAACUCAUUCAUTT	AUGAAUGAGUUGCGAAGGCTT
siRNA-3	CAGGAGCAUUUCCCAACAUTT	AUGUUGGGAAAUGCUCCUGTT
siNC	UUCUCCGAACGUGUCACGUTT	ACGUGACACGUUCGGAGAATT

Primer	F primer (5'-3')	R primer (5'-3')
CACNA1G-AS1	TTGTTGGCCGGAGCACTAAT	AGTGAAGCAGGAAGGAACCG
GAPDH	GTCAACGGATTTGGTCTGTATT	AGTCTTCTGGGTGGCAGTGAT

After RNA extraction and reverse transcription, real-time qPCR analysis was performed using an ABI 7500 instrument to evaluate the expression level of lncRNA CACNA1G-AS1 in cells and tissue based on the kit from TAKARA (Japan). The primer sequences are listed in Table 1.

Transfected SKOV-3 and A2780 cells were made into cell suspensions and then transferred to a 96-well plate. The old culture media was removed, and 10 µl cell counting kit‐8 solution (MedChemExpress, China) with 90 µl media was added to each well for an additional two hours on days one–four. At a wavelength of 450 nm, the OD value of each well was detected by a spectrophotometer (Thermo Scientific).

### Statistical analysis

We performed and visualized statistical analysis by using R packages (v4.0.2), TBtools and GraphPad Prism (v8.0). Kaplan–Meier (K-M) survival analysis of risk groups and clinicopathology subgroups were performed by the log-rank test to draw a survival curve. Wilcoxon test was utilized to compare difference between two groups. The experiments were conducted in triplicate, and each experiment was repeated three times. The experimental data were analyzed by Student’s t-test or one-way analysis of variance (ANOVA). P < 0.05 was considered statistically significant.

### Results

**Fig. 1:**
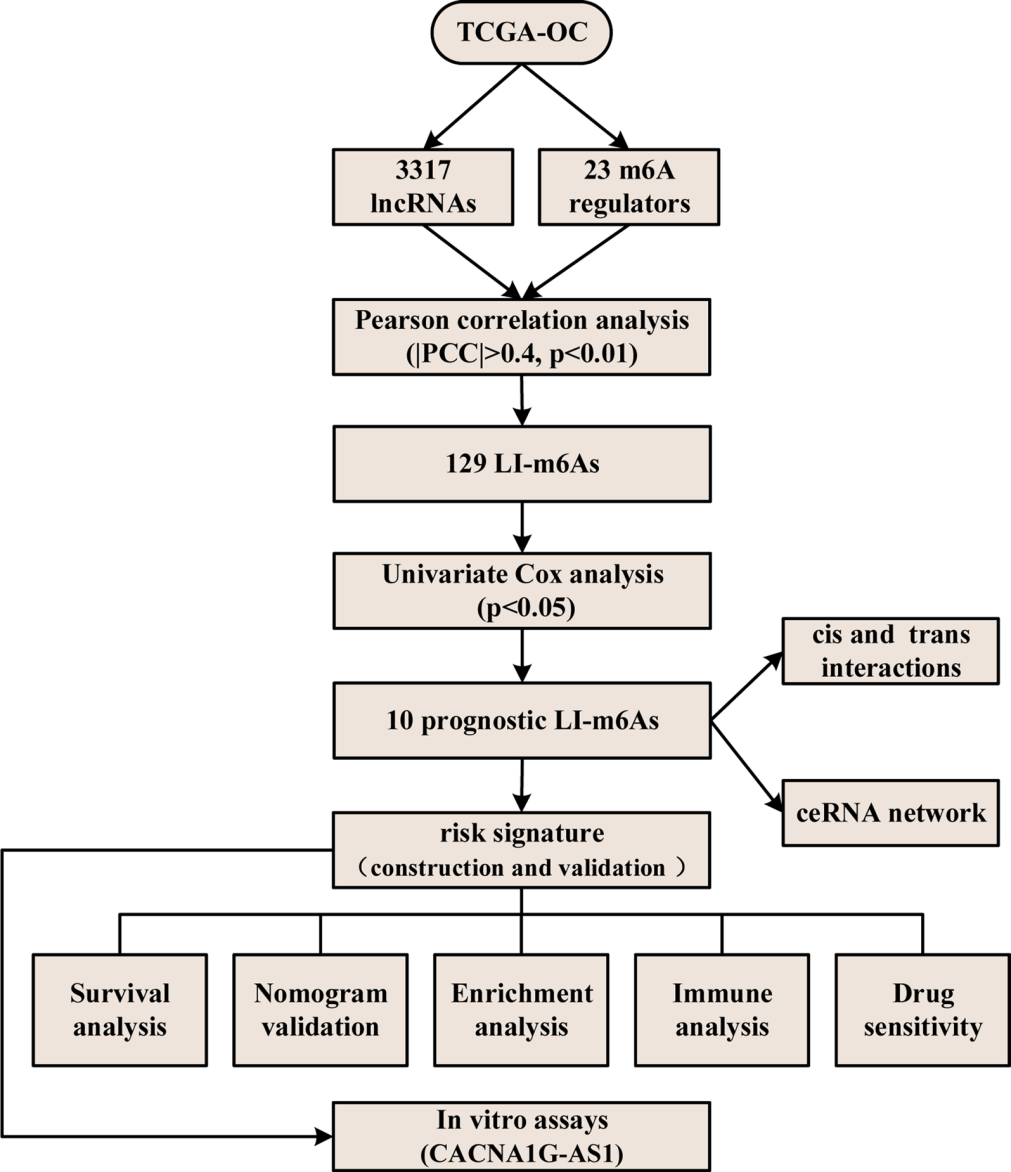
Flow diagram of our study

To facilitate the understanding of our entire study, we created a flowchart, which is shown in Fig. 1.

### Identification of significant prognostic LI-m6As

**Fig. 2:**
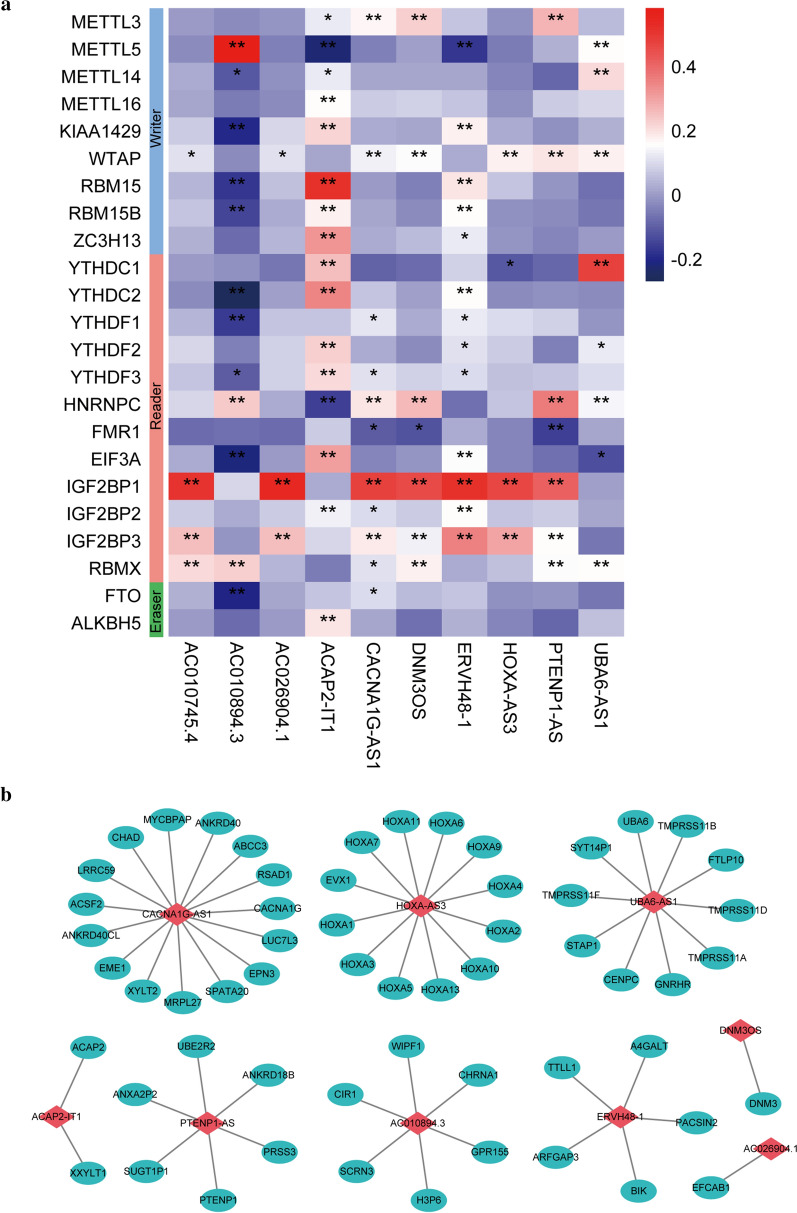
Identification of significant prognostic LI-m6As. **a** Correlations between 23 regulators and 10 significant prognostic LI-m6As. *p < 0.05 and **p < 0.01. **b** lncRNA-nearby mRNA interaction networks. Red rhombus: LI-m6As; blue ellipses: nearby mRNAs

A lncRNA that significantly correlated with one or more of the 23 m6A regulators was identified as LI-m6As (|PCC|> 0.4 and p < 0.01), and 10 significant prognostic LI-m6As were identified after univariate Cox regression analysis on the 129 acquired LI-m6As. As shown in the heatmap (Fig. 2a), seven significant prognostic LI-m6As (AC010745.4, AC026904.1, CACNA1G-AS1, DNM3OS, ERVH48-1, HOXA-AS3, and PTENP1-AS) were markedly correlated with IGF2BP1 (reader). In addition, AC010894.3, ACAP2-IT1, and UBA6-AS1 were correlated with METTL5 (writer), RBM15 (writer), and YTHDC1 (reader), respectively.

**Fig. 3:**
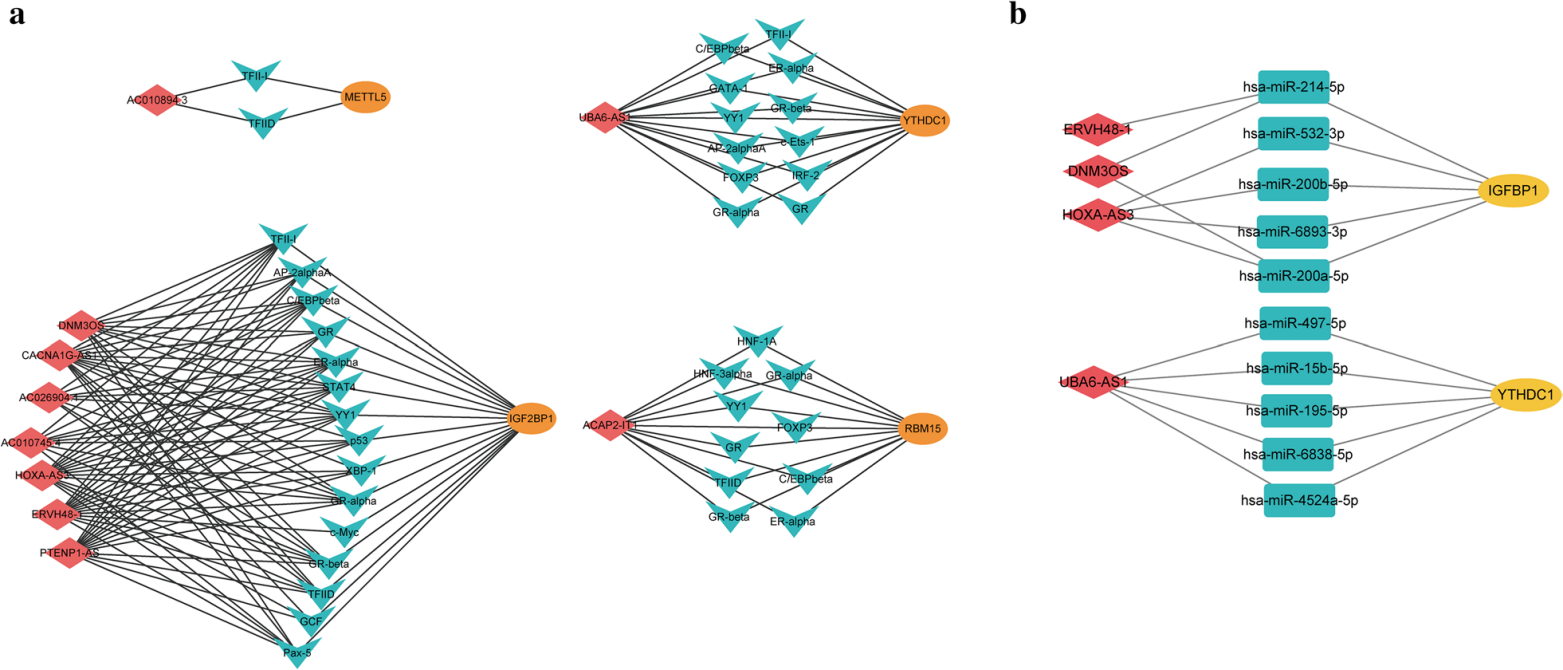
Network based on trans interactions and ceRNA. **a** Trans interaction for lncRNA-TF-mRNA relationship pairs. **b** ceRNA for lncRNA-miRNA-mRNA relationship pairs. Blue arrowheads: TFs; red rhombus: LI-m6As; yellow ellipses: m6A regulators; blue rectangles: miRNAs

A total of 58 LI-m6As-nearby-targeted mRNAs pairs, including nine of the ten LI-m6As, were detected. There were no nearby targeted mRNAs of AC010745.4 (Fig. 2b). As for trans interactions, we searched for the TFs overlapping with LI-m6As and m6A regulators and a total of 108 lncRNA-TF-mRNA relationship pairs were found (Fig. 3a, Additional file 1: Table S1). Interestingly, p53 may regulate m6A factors and LI-m6As through trans interactions (p53-IGF2BP1-AC010745.4/CACNA1G-AS1/ERVH48-1/HOXA-AS3/PTENP1-AS). On the basis of ten lncRNA-m6A pairs, we further found four of them included in the lncRNA-miRNA-mRNA relationship pairs and the top one lncRNA-m6A pair with most miRNAs was UBA6-AS1-YTHDC1(Fig. 3b). These findings may provide insights into the regulatory mechanisms of lncRNAs correlated with m6A-regulatory genes.

### Construction and validation the prognostic signature

**Fig. 4:**
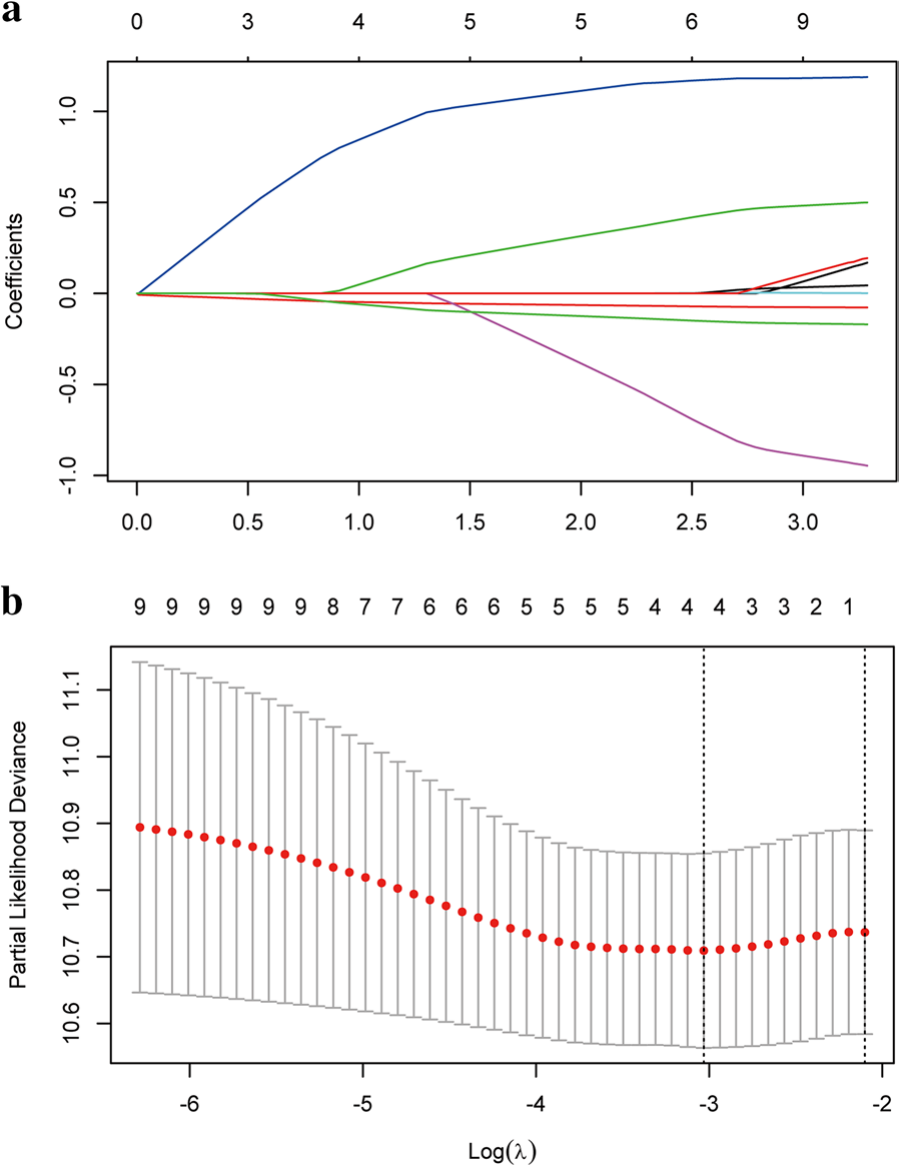
**a** LASSO Cox analysis of 10 significant prognostic LI-m6As. **b** λ selection diagram. The two dotted lines indicated two particular values of λ. The left side was λ_min_ and the right side was λ_1se_. The λ_min_ was selected to build the model for accuracy in our study

**Fig. 5:**
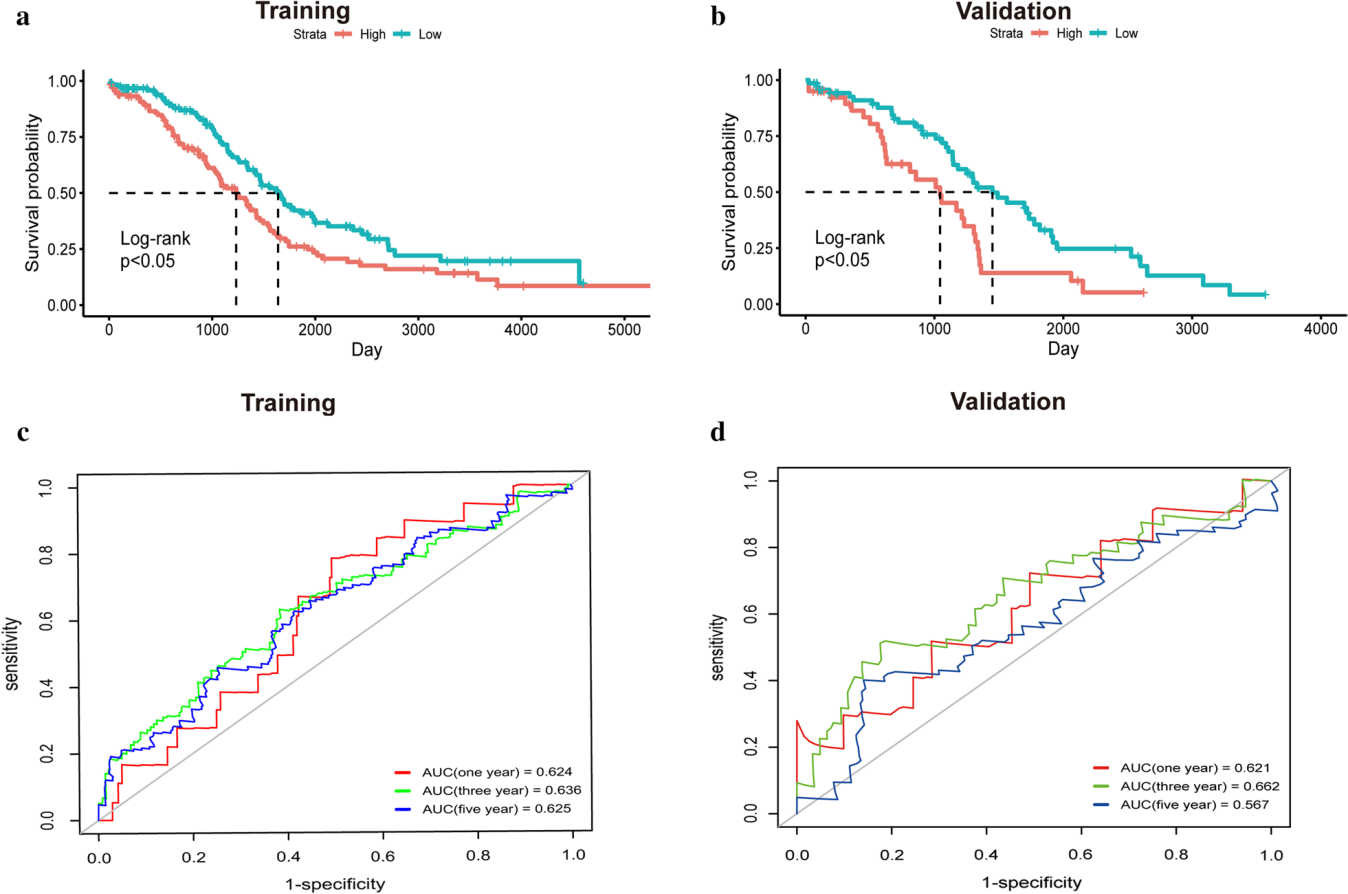
Construction and validation of the prognostic signature. **a, b** The K-M survival curves of two risk groups based on the risk model in the training cohort **(a)** and validation cohort **(d)**. **c**, **d** The ROC curves for predicting 1-, 3-, and 5-year survival in in the training cohort **(c)** and validation cohort **(d)**

A LI-m6A prognostic signature was constructed according to the LASSO Cox analysis of 10 significant prognostic LI-m6As. The λ selection diagram is shown in Fig. 4a, b. λ between λ_min_ and λ_1se_ were considered appropriate. The model constructed by λ_1se_ was the simplest, that was, it used a small number of genes, while λ_min_ had a higher accuracy rate and used a larger number of genes. The λ_min_ was selected to build the model for accuracy in our study. Patients in the training and validation cohorts were divided into low- or high-risk subgroups based on the median of RSs. The K–M survival curves of risk groups revealed that OS in the high-risk group was markedly lower than that in the low-risk group in both cohorts (Fig. 5a, b). The ROC curves proved that the risk model signature based on four m6A-RLs was accurate in predicting the OS of OC patients (Fig. 5c, d).

**Fig. 6:**
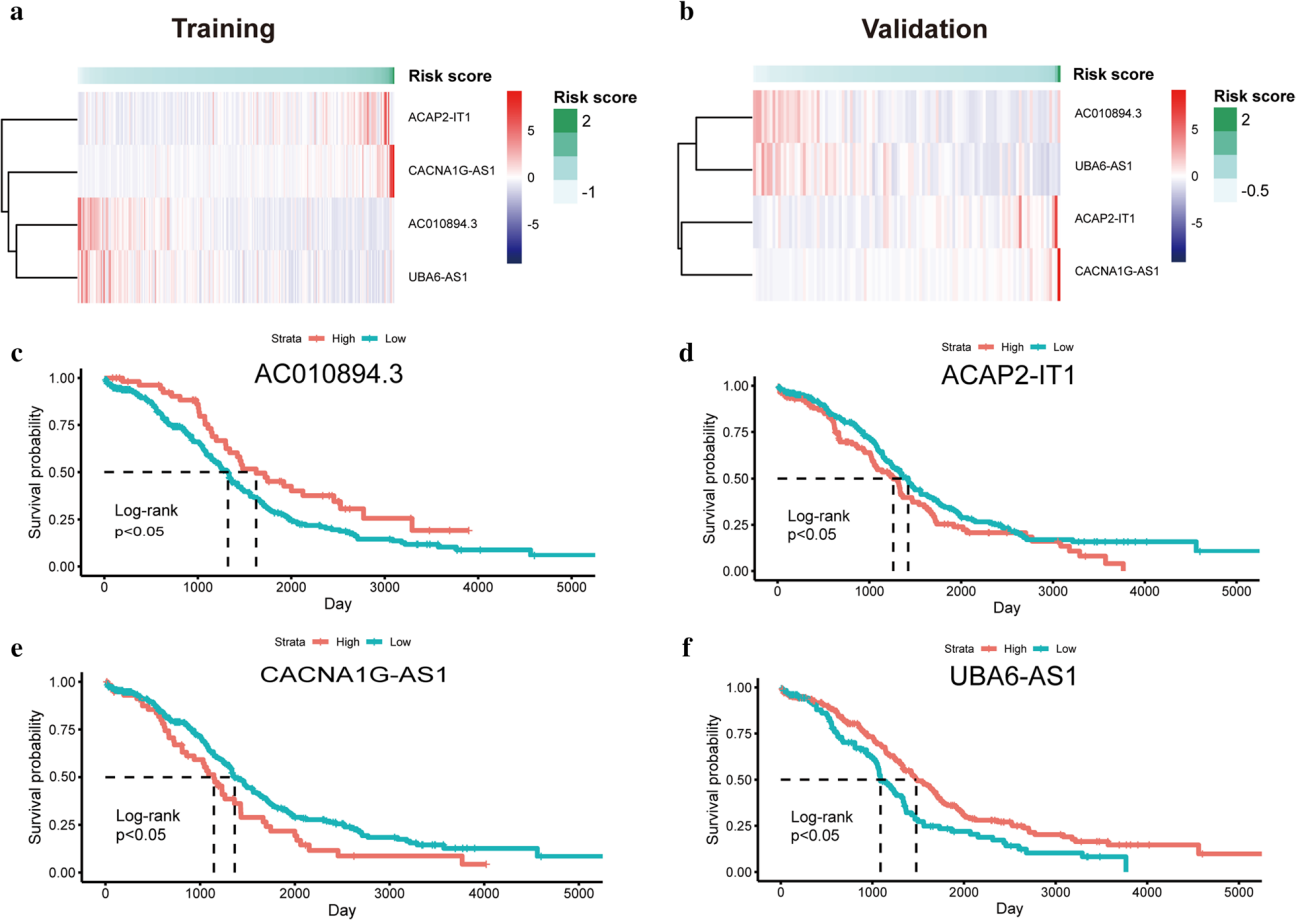
Prognostic analysis of the four LI-m6As. **a**, **b** Heatmap of the expression levels of the four m6A-RLs with RSs in the training **(a)** and validation cohort **(b)**. **c**-**f** The K-M survival curves of LI-m6A AC010894.3 **(c)**, ACAP2-IT1 **(d)**, CACNA1G-AS1 **(e)**, and UBA6-AS1 **(f)**

Four LI-m6As with corresponding coefficients and HR values (Table 2), namely, AC010894.3, ACAP2-IT1, CACNA1G-AS1, and UBA6-AS1, were generated to calculate RSs.. A heatmap of the associations between the expression levels of the four LI-m6As and RSs showed that the expression of ACAP2-IT1 and CACNA1G-AS1 increased with increasing RS, whereas the expression of AC010894.3 and UBA6-AS1 decreased with increasing RS in both the training and validation cohorts (Fig. 6a, b). The K–M survival curves confirmed that lower expression of ACAP2-IT1 and CACNA1G-AS1 and higher expression of AC010894.3 and UBA6-AS1 were associated with better OS of OC patients (Fig. 6c-f). Hence, ACAP2-IT1 and CACNA1G-AS1 were risk factors, while AC010894.3 and UBA6-AS1 were protective factors.

**Table 2:** The coefficient and HR value of the four m6A-related lncRNAs

lncRNA	Coefficient	HR	HR 95%CI (lower)	HR 95%CI (upper)
AC010894.3	− 0.0710905	0.93137761	0.851050332	1.01928667
ACAP2-IT1	0.37794931	1.45928897	0.802982948	2.652016842
CACNA1G-AS1	1.26113752	3.52943399	1.681058447	7.410155391
UBA6-AS1	− 0.163757	0.84894828	0.65798919	1.095326775

### Nomogram model construction and visualization

**Fig. 7:**
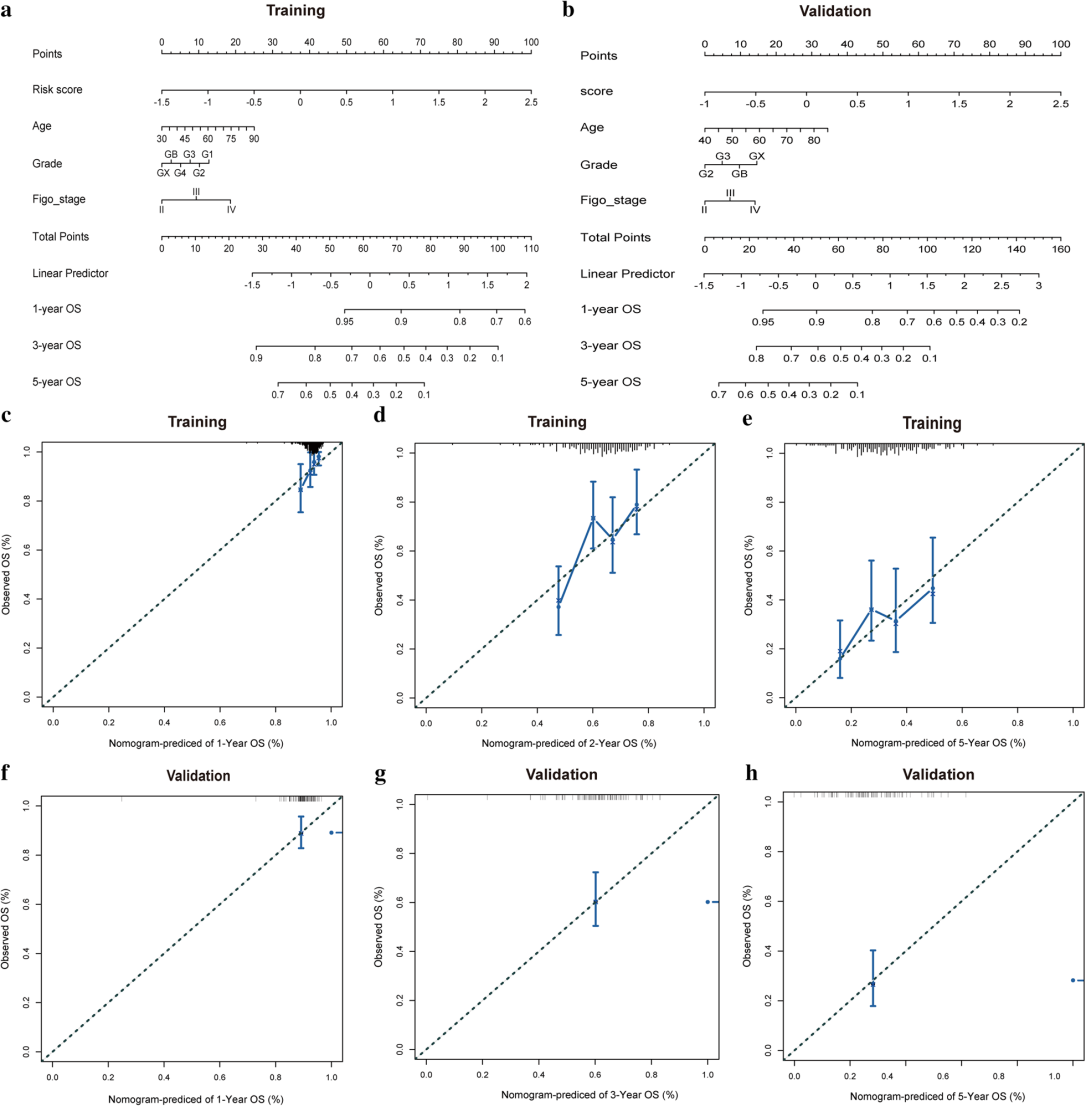
Nomogram model construction and visualization. **a**, **b** The Nomogram model based on risk score and clinical features for the training **(a)** and validation **(b)** cohort. **c-h** The calibration plots of the nomogram for predicting the probability of OS at 1 **(c, f)**, 3 **(d, g)**, and 5 **(e, h)** years in the training cohort **(c–e)** and validation cohort **(f–h)**

Univariate and multivariate Cox regression analyses of clinicopathology and the LI-m6A risk model showed that the signature was an independent risk factor for OC patients in the training and validation cohorts (Table 3). A nomogram was further constructed based on risk score, age, grade, and Figo state to predict the prognosis of OC (Fig. 7a, b). A calibration diagram was also used to verify the prediction ability of risk score for 1-, 3- and 5-year of survival (Fig. 7c–h). In the calibration plots, the blue solid line was the prediction for survival, and the diagonal dotted line was the actual survival. The closer the solid line was to the dotted line, the better the prediction ability was.

**Table 3:** Univaraite and multivariate Cox analysis of risk score and clinicopathology

Variables	Univariate			Multivariate		
	Coefficient	Hazard ratio	P	Coefficient	Hazard ratio	P
Training cohort						
Risk score	1	2.718 (1.741–4.244)	< 0.001	1.071	2.917 (1.549–5.492)	0.001
Age	0.02	1.020 (1.004–1.037)	0.015	0.018	1.018 (1.001–1.035)	0.037
Grade	0.066	1.068 (0.787–1.448)	0.673	− 0.109	0.896 (0.637–1.263)	0.532
Figo_stage	0.359	1.432 (0.981–2.089)	0.062	0.398	1.489 (0.980–2.262)	0.062
Validation cohort						
Risk score	0.949	2.583 (1.335–4.994)	0.005	0.853	2.347 (1.189–4.633)	0.014
Age	0.023	1.023 (0.997–1.05)	0.087	0.023	1.023 (0.996–1.051)	0.096
Grade	0.242	1.274 (0.752–2.159)	0.368	0.146	1.157 (0.646–2.073)	0.624
Figo_stage	0.051	1.052 (0.679–1.630)	0.819	0.211	1.235 (0.750–2.033)	0.407

### Clinicopathology analysis

**Fig. 8:**
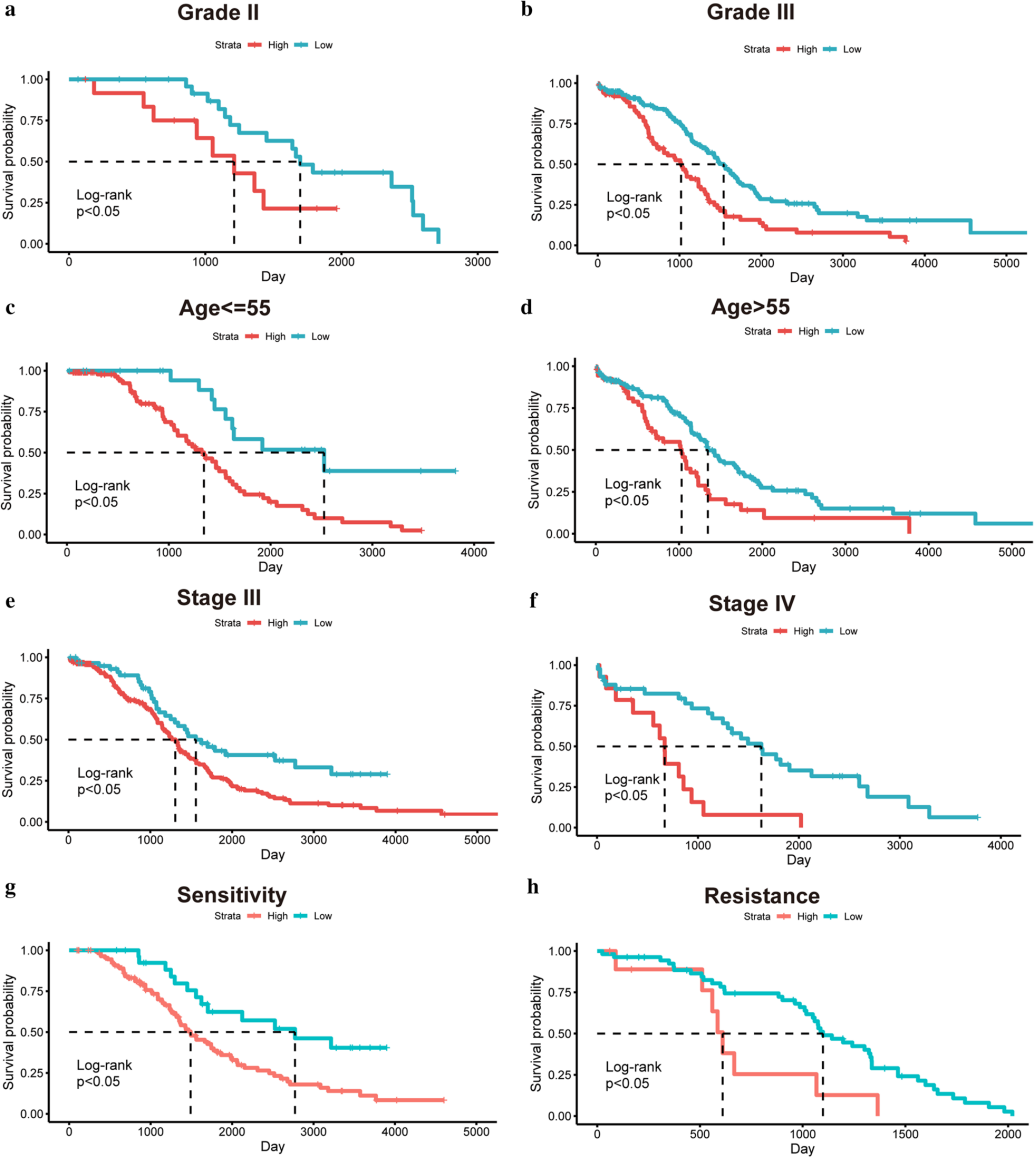
The K-M survival curves of several clinicopathological characteristics based on the risk model. **a** Grade II. **b** Grade III. **c** Age <  = 55. **d** Age > 55. **e** Stage III. **f** Stage IV. **g** Platinum sensitivity. **h** Platinum resistance

We carried out clinicopathological analysis to evaluate the prognostic capacity of the LI-m6As prognostic signature. Several clinicopathological characteristics, such as WHO grade I, grade IV and FIGO stage I, were excluded due to the small sample size (< 10 patients). The results showed that the OS of the clinicopathology subgroups, such as Grade II, Grade III, age <  = 55 or > 55 years old, Stage III, Stage IV, platinum sensitivity and platinum resistance, were significantly different in the low- or high-risk group (Fig. 8a-h). Potentially, the p value of the Stage II subgroup was indistinctive because of insufficient sample size (Additional file 2: Fig. S1).

### Biofunctional analysis of the risk groups

**Fig. 9:**
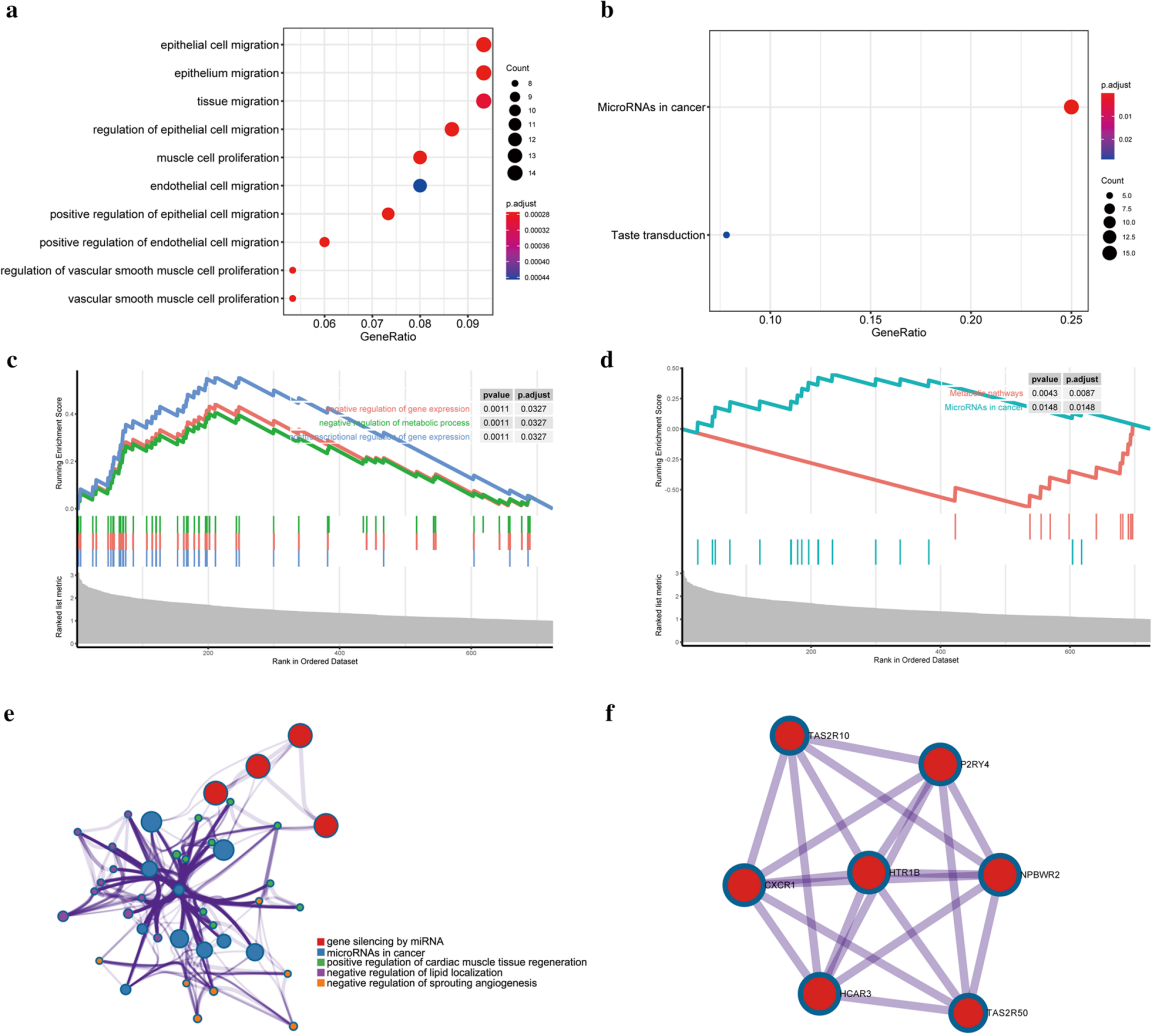
Functional analysis. **a**, **b** Significantly enriched GO terms **(a)** and KEGG pathways **(b)** of differentially expressed genes (DEGs). The color scale represented p value and the circle size indicated count. **c**, **d** Gene set enrichment analysis (GSEA) of GO **(c)** and KEGG **(d)**. The crest on the left represents the high expression of the enriched genes in the high-risk group, while the crest on the right is the opposite. **e** Cluster ID. Nodes with the same cluster ID are typically close to each other and the same color indicates the same cluster ID. **f** The key module of PPI network. Seven hub genes were included in the interaction network. Circles indicate the genes in the PPI network, and the connection indicates the potential interaction between different mRNAs

A total of 1581 DEGs between the risk subgroups were identified. The GO biological process analysis found that DEGs were mainly enriched in migration and proliferation, such as epithelial cell migration and vascular smooth muscle cell proliferation (Fig. 9a). Moreover, the performed KEGG pathway analysis revealed that the DEGs were mainly enriched in microRNAs in cancer and taste transduction (Fig. 9b). GSEA of GO indicated that gene sets enriched in ‘negative regulation of gene expression’, ‘negative regulation of metabolic process’, and ‘posttranscriptional regulation of gene expression’ were highly expressed in the high-risk group (Fig. 9c). GSEA of KEGG showed that genes enriched in ‘MicroRNAs in cancer’ were highly expressed the in high-risk group, while genes enriched in ‘Metabolic pathways’ were expressed at low levels in the high-risk group (Fig. 9d). The enriched GO terms and KEGG pathways were further annotated from “Metascape” website (Fig. 9e). The key module of the PPI network discerned by MCODE were shown in Fig. 9f. Seven hub genes were included in the interaction network.

### Immune microenvironment and checkpoint

**Fig. 10:**
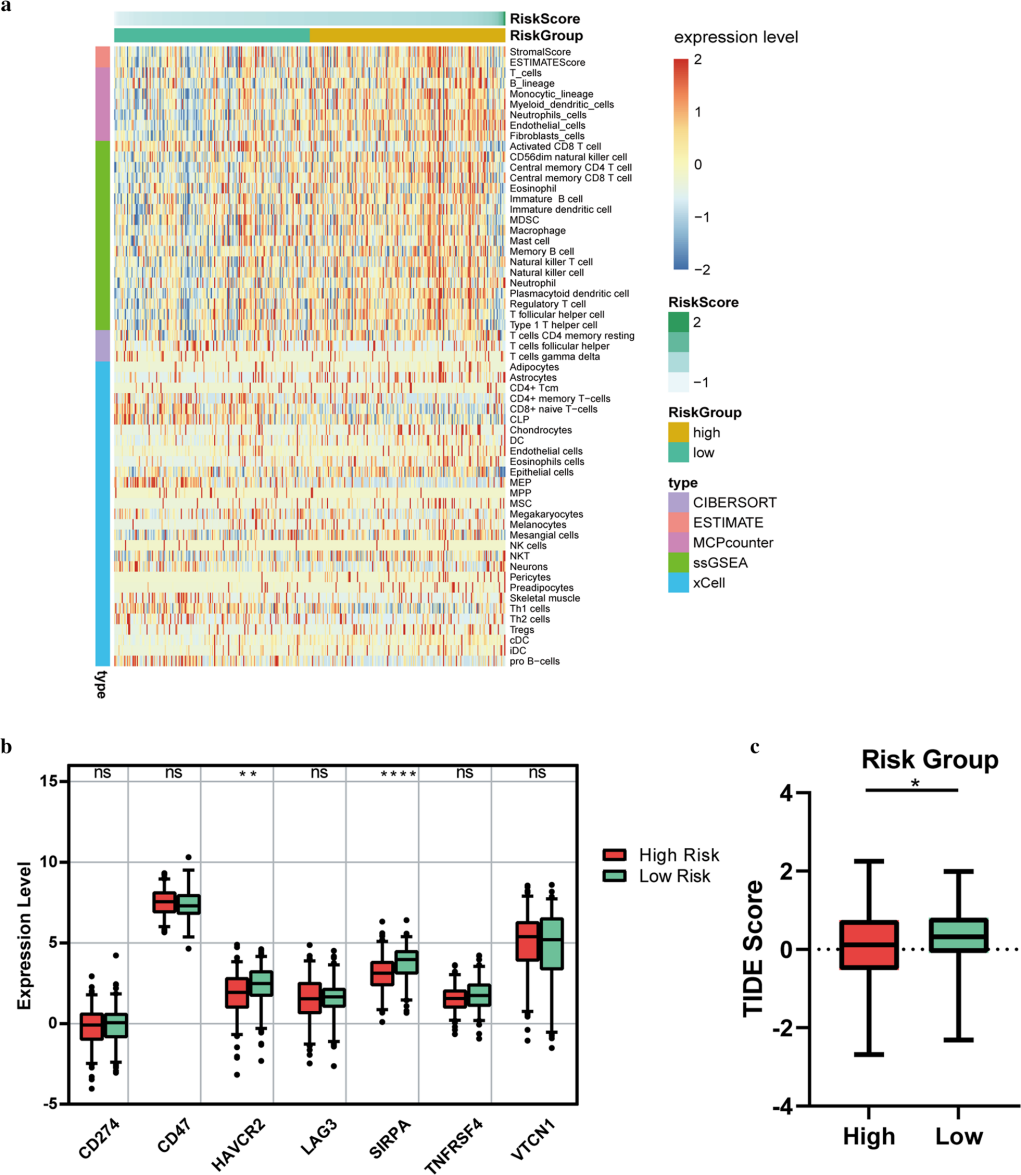
Immune microenvironment and checkpoint. **a** Heatmap of immune microenvironment revealed that a total of 59 immune cells and stromal score had significant differences between the two risk groups. **b** Expression of seven immune checkpoint genes between high and low-risk group. HAVCR2 and SIRPA had higher expressions in the low-risk group. **c** TIDE scores in the low-risk group were higher than those in the high-risk group. Data are shown as means ± S.D. *ns* not significant, *p < 0.05, **p < 0.01, ***p < 0.001, ****p < 0.0001

According to five algorithms (Estimate, ssGSEA, Cibersort, MCPcounter and xCell), Stromal Score, Immune Score, ESTIMATE Score, and relative infiltration abundance of immune cells and stromal cells of each sample were estimated respectively. The results revealed that a total of 59 microenvironment cells and Stromal score had significant differences between the two groups (Fig. 10a, Additional file 3: Table. S2). Among them, Neutrophils cells, Fibroblasts cells, and Endothelial cells were the top 3 immune cells significantly enriched in the high-risk group. Given the importance of immune checkpoints in cancer treatment, the expressions of seven checkpoint genes were compared. We found that HAVCR2 and SIRPA had higher expressions in the low-risk group (Fig. 10b). In Fig. 10c, OC patients in low-risk group exhibited higher TIDE scores than those in high-risk group, indicating that OC patients with lower RSs were more sensitive to ICB therapy.

### Sensitivity of chemotherapy drug

**Fig. 11:**
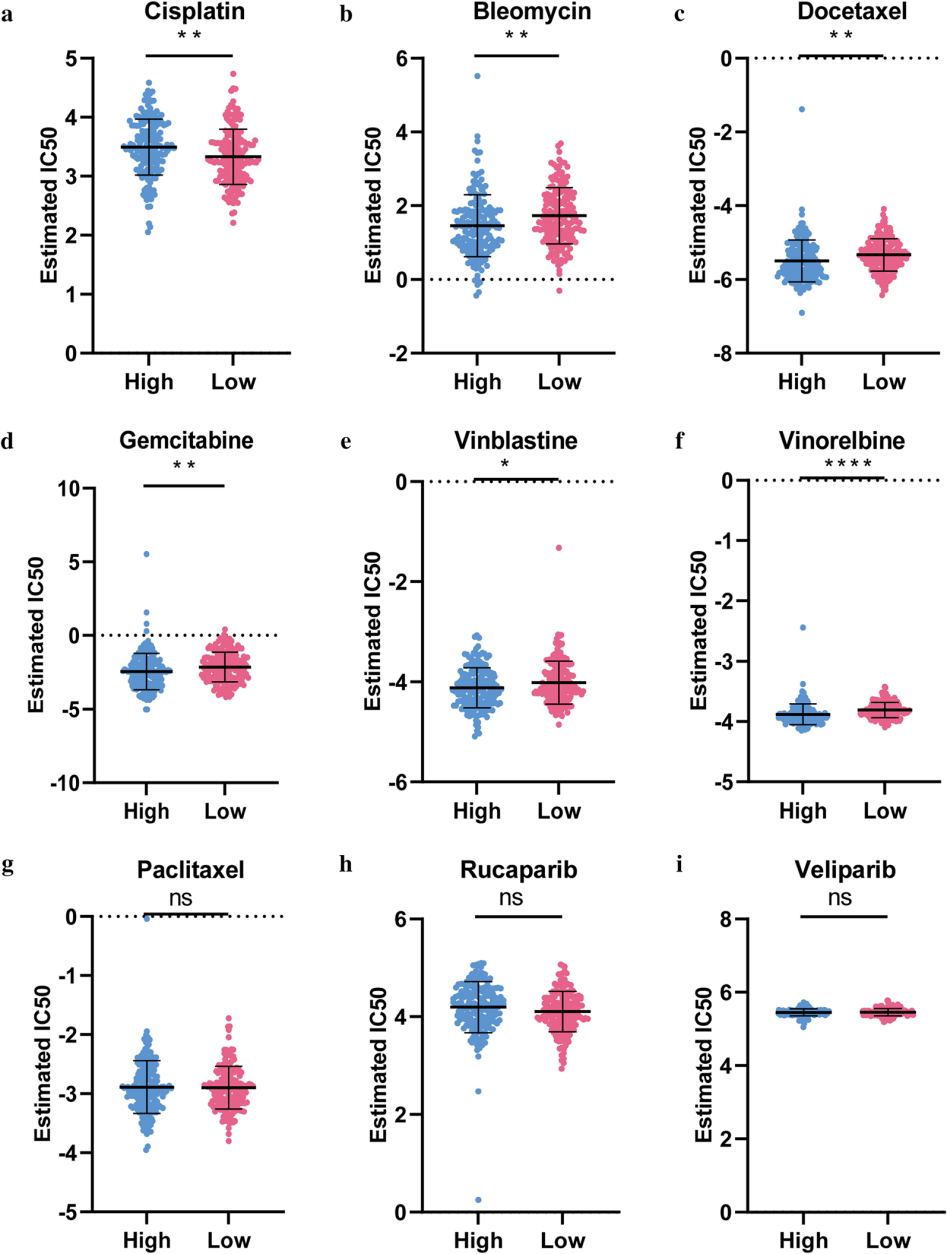
Sensitivity of chemotherapy drug. **a**-**i** Difference in the estimated IC50 levels of Cisplatin **(a)**, Bleomycin **(b)**, Docetaxel **(c)**, Gemcitabine **(d)**, Vinblastine **(e)**, Vinorelbine **(f)**, Paclitaxel **(g)**, Rucaparib **(h)** and Veliparib **(i)**. Data are shown as means ± S.D. *ns* not significant, *p < 0.05, **p < 0.01, ***p < 0.001, ****p < 0.0001

The IC50 levels of 135 drugs in OC patients were quantified and significant differences in 87 chemotherapy drugs were found between the risk groups (Additional file 4: Table. S3). Figure 11 exhibited the results of nine commonly used chemotherapeutic agents for OC. Our data showed that the IC50 levels of Cisplatin (Fig. 11a) were significantly higher in high-risk group than that in low-risk group. Inversely, the IC50 levels of Bleomycin (Fig. 11b), Docetaxel (Fig. 11c), Gemcitabine (Fig. 11d), Vinblastine (Fig. 11e) and Vinorelbine (Fig. 11f) were significantly lower in high-risk group than that in low-risk group, indicating that the OC patients in the high-risk group were more sensitive to these drugs. However, the sensitivity of the two risk groups to Paclitaxel (Fig. 11g), Rucaparib (Fig. 11h) and Veliparib (Fig. 11i) did not show a significant difference.

### In vitro assays

**Fig. 12:**
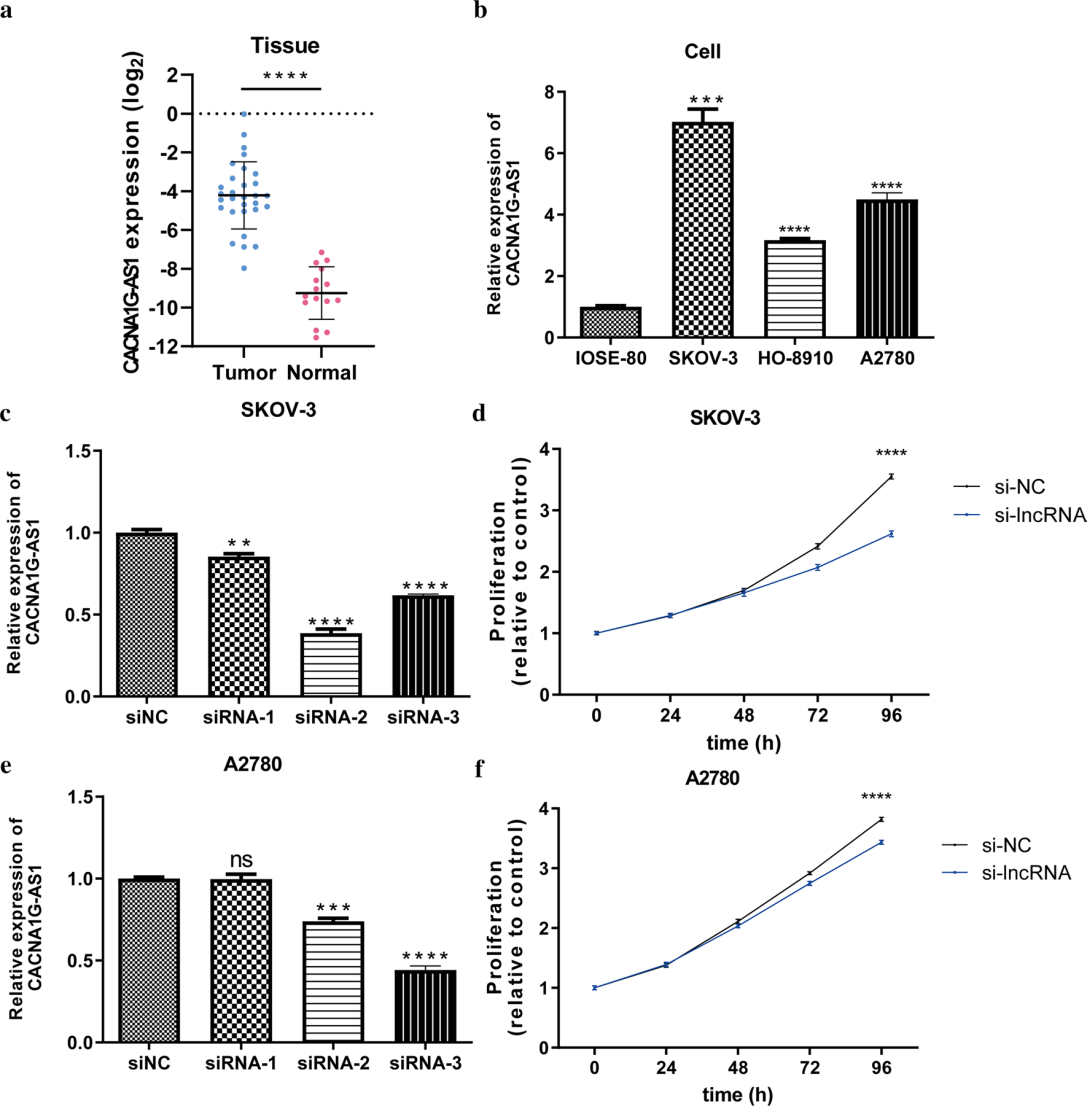
Cell function assays. **a, b** Real-time qPCR analysis detecting relative expression of lncRNA CACNA1G-AS1 in 30 OC tissues **(a)** and three OC cell lines **(b)**. **c** The transfection efficacities of three siRNAs targeting CACNA1G-AS1 in SKOV-3 cells. **d** CCK-8 assays were performed after CACNA1G-AS1 was inhibited in SKOV-3. **e** The transfection efficacities of three siRNAs targeting CACNA1G-AS1 in A2780. **f** CCK-8 assays were performed after CACNA1G-AS1 was inhibited in A2780. Data are shown as means ± S.D. *ns* not significant, *p < 0.05, **p < 0.01, ***p < 0.001, ****p < 0.0001

lncRNA CACNA1G-AS1 was filtered as the candidate molecule to perform cell function assays. Real-time qPCR analysis indicated that CACNA1G-AS1 was significantly upregulated in 30 OC tissues and three OC cell lines (Fig. 12a, b). The transfection efficiencies of three siRNAs targeting CACNA1G-AS1 were detected, which revealed that siRNA-2 presented the highest transfection efficiency in SKOV-3 cells (Fig. 12c) and that siRNA-3 presented the highest transfection efficiency in A2780 cells (Fig. 12e). The CCK-8 assay showed that the viability of SKOV-3 (Fig. 12d) and A2780 cells (Fig. 12f) was suppressed after transfection of the corresponding siRNAs.

## Discussion

As the most common internal modification of mRNAs in all higher eukaryotes, m6A has been proven to be abnormally expressed in a variety of tumors, and plays an important role in the regulation of a series of malignant biological behaviors such as cell proliferation, invasion and metastasis [2]. Previous studies have reported that several lncRNAs regulate the occurrence and development of cancer via m6A modification. For example, lncRNA RP11-138 J23.1 regulated by m6A positively induced the epithelial mesenchymal transition (EMT) of colorectal cancer cells [38]. Oncogenic lncRNA XIST is inhibited by METTL14 (writer) in colorectal cancer [39]. The lncRNA DANCR and IGF2BP2 (reader) have been proven to synergistically promote the pathogenesis of pancreatic cancer [40]. It has been unveiled that LINC00942 serves as an oncogene by promoting METTL14-mediated m6A methylation in breast cancer [41]. lncRNA GAS5 negatively regulated by YTHDF3 (reader) was demonstrated to be a tumor-suppressor in colorectal cancer [42]. LINRIS was proven to promote carcinogenesis of colorectal cancer by serving as a stabilizer of IGF2BP2 (reader) [43]. LNCAROD is aberrantly expressed and has been identified as an oncogenic lncRNA due to dysregulation of m6A modification [44]. The abovementioned evidence shows that m6A is modified in the process of lncRNA biological function. In addition, lncRNA can also regulate m6A modification. These are all part of the complex regulatory network of tumors. As a new discovery in scientific research, we believe that in-depth study of the biological mechanism of mutual regulation between lncRNAs and m6A will become a hot spot for discovering prognostic markers or therapeutic targets for malignant tumors. Nonetheless, lncRNAs involved in m6A regulation in OC are still unknown.

Four LI-m6As, namely, AC010894.3, ACAP2-IT1, CACNA1G-AS1, and UBA6-AS1, were included in our identified prognostic signature. They were correlated with METTL5 (writer), RBM15 (writer), IGF2BP1 (reader), and YTHDC1 (reader), respectively. Among the four m6A regulators, METTL5 was confirmed to serve as the enzyme for the m6A modification of 18S rRNA, thereby promoting breast cancer cell growth [45]. RBM15 was identified as a regulator that binds to METTL3 and WTAP and directs these two proteins to specific RNA sites for m6A modification [2]. Studies have shown that lncRNAs participate in mediating the occurrence and progression of cancer by targeting IGFBP1, thus becoming a new therapeutic target for cancer [46]. The m6A level of lncRNA pncRNA-D was recognized by YTHDC1 [47]. For the LI-m6As we identified, a study has shown that UBA6-AS1 serves as a malignant gene in glioblastoma by competitively binding to miR-7648. Previous studies have demonstrated that lncRNA CACNA1G-AS1 enhances the multiplication capacity via the miR-2392/C1orf61 pathway in hepatocellular cancer [49]. CACNA1G-AS1 promoted the proliferation and invasiveness of colorectal cancer by inhibiting the expression of p53 [50]. CACNA1G-AS1 exerts its malignant function by HNRNPA2B1 in non-small-cell lung cancer [51]. In our research, CACNA1G-AS1 showed high expression and may regulate proliferation, which would fill the gap in the OC literature. Our findings are probably worthy of further study by subsequent researchers who concentrate on the mechanism of m6A-related lncRNAs in OC.

We further explored the differences in immune microenvironment, immune checkpoint, chemotherapy drug sensitivity between the two risk groups. Our data showed that OC patients in the low-risk group may be more sensitive to Cisplatin and ICB therapy compared to the high-risk group, while patients in the high-risk group were more sensitive to Bleomycin, Docetaxel, Gemcitabine, Vinblastine and Vinorelbine. Findings of our study uncovered potential biomarker and therapeutic target for the risk model based on the LI-m6As.

Our study still has some limitations. First, there were only 30 OC patients without OS in our cohort, hence, more time and more samples are needed for follow-up. Second, cell function assays of CACNA1G-AS1 were preliminary and need further investigation to provide a better understanding. Definitively, the number of OC samples in TCGA is very limited; hence, more independent data sets are needed to validate our identified LI-m6As.

### Conclusions

We found four lncRNAs (AC010894.3, ACAP2-IT1, CACNA1G-AS1, and UBA6-AS1) involved in m6A regulation and that have significant prognostic value in OC. Furthermore, the predictive signature based on the four lncRNAs can independently predict the therapeutic value of OC patients by combining molecular signatures and clinical characteristics. Furthermore, CACNA1G-AS1 was preliminarily identified as a malignant lncRNA through in vitro experiments.

## Supplementary Information

**Additional file 1: Table S1.** Trans-regulation lncRNA-TF-mRNA.

**Additional file 2: Figure S1.**The K-M survival curve of Stage II based on the risk model. The p value was indistinctive.

**Additional file 3: Table S2.** Difference in Immune microenvironment.

**Additional file 4: Table S3.** Drugs with significant difference in sensitivity.

## Data Availability

The RNA sequencing profiles are able to be gained from The Cancer Genome Atlas (TCGA) (https://toil.xenahubs.net). Further inquiries can be directed to the corresponding author.

## Abbreviations

DEG: Differentially expressed genes
GO: Gene ontology
GRCh38: Genome Reference Consortium Human Build 38
GSEA: Gene Set Enrichment Analysis
HR: Hazard ratio
K-M: Kaplan–Meier
LASSO: Least absolute shrinkage and selection operator
LI-m6As: LncRNA involved in m6A regulation
lncRNA: Long noncoding RNA
m6A: N6-methyladenosine
OC: Ovarian cancer
OS: Overall survival
PPI: Protein–protein interaction
RS: Risk score
TCGA: The Cancer Genome Atlas
TIDE: Tumor Immune Dysfunction and Exclusion
