# Supplementary figures and images for: Identification and validation of lncRNAs involved in m6A regulation for patients with ovarian cancer
Source: Cancer Cell Int. 2021 Jul 8;21:363. doi: 10.1186/s12935-021-02076-7 (PMC8268297; doi:10.1186/s12935-021-02076-7)

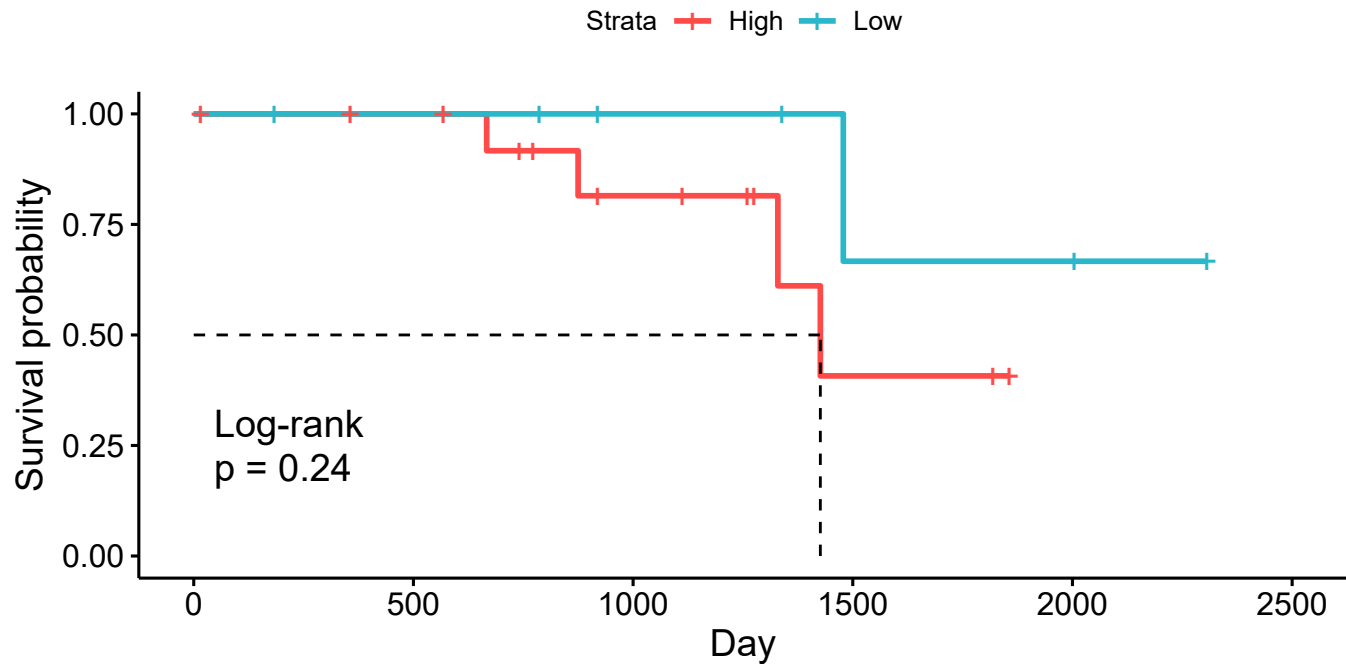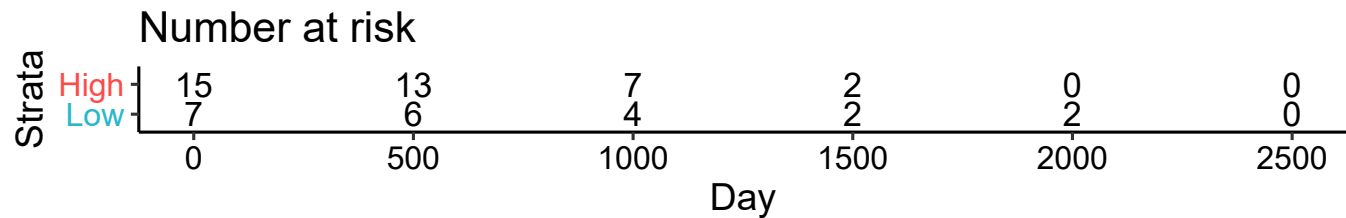

Supplement: Supplementary file 2 — Additional file 2: Figure S1.The K-M survival curve of Stage II based on the risk model. The p value was indistinctive. [file 12935_2021_2076_MOESM2_ESM.pdf]
